# Efficacy and Tolerability of Pazopanib in Elderly Patients with Advanced Soft Tissue Sarcoma: A Multicentre Real-World Study from Turkey

**DOI:** 10.3390/jcm15124803

**Published:** 2026-06-20

**Authors:** Mehmet Mutlu Kidi, Harun Muğlu, Mustafa Karaağaç, Sinan Koca, Oguz Kara, Ahmet Bilici, Ertugrul Bayram

**Affiliations:** 1 Department of Medical Oncology, Faculty of Medicine, Cukurova University, Adana 01330, Turkey; 2 Department of Medical Oncology, Istanbul Medipol University, Istanbul 34214, Turkey; 3 Department of Medical Oncology, VM Medical Park Samsun Hospital, Samsun 55200, Turkey; 4 Department of Medical Oncology, Göztepe Prof. Dr. Süleyman Yalçın City Hospital, Istanbul 34722, Turkey

**Keywords:** soft tissue sarcoma, pazopanib, elderly, geriatric oncology, real-world evidence, progression-free survival, overall survival

## Abstract

**Background**: Soft tissue sarcomas (STS) disproportionately affect older adults, yet patients aged ≥65 years remain markedly underrepresented in pivotal trials, limiting evidence on pazopanib in this population. We aimed to characterise the real-world efficacy and safety of pazopanib in elderly patients with advanced STS. **Methods**: This multicentre retrospective cohort study included consecutive patients aged ≥65 years with locally advanced unresectable or metastatic STS who received pazopanib between July 2010 and June 2022 at four tertiary Turkish oncology centres. The primary endpoint was progression-free survival (PFS); secondary endpoints were overall survival (OS) and the safety profile. **Results**: A total of 109 patients (median age, 70 years; 50.5% female; 48.6% with Eastern Cooperative Oncology Group [ECOG] performance status ≥ 2) were analysed. The objective response rate was 11.0% (95% CI, 5.8–18.4), and the disease control rate was 45.9%. Median PFS was 4.11 months (95% CI, 3.25–4.47), and median OS was 7.85 months (95% CI, 6.91–9.00) over a median follow-up of 17.6 months. PFS showed a borderline difference across age tertiles (log-rank *p* = 0.078), whereas a marked monotonic OS gradient was observed (9.00, 7.86, and 5.71 months for ages 65–69, 70–74, and ≥75 years, respectively; *p* < 0.001). In age-stratified multivariable Cox analysis, ECOG ≥ 2 (adjusted hazard ratio [aHR], 1.68; 95% CI, 1.01–2.80; *p* = 0.045) and female sex (aHR, 1.66; 95% CI, 1.02–2.72; *p* = 0.043) were independently associated with shorter OS. Grade ≥ 3 treatment-emergent adverse events occurred in 27.5% of patients, most commonly hypertension. Because only the single most clinically prominent treatment-emergent adverse event per patient was recorded, these figures represent a conservative, non-cumulative estimate of toxicity. No treatment-related deaths occurred. **Conclusions**: Pazopanib retains clinically meaningful activity in unselected patients aged ≥65 years with advanced STS. Performance status, rather than chronological age, is the dominant predictor of overall survival and should guide treatment decisions in this population.

## 1. Introduction

Soft tissue sarcomas (STS) are a heterogeneous group of mesenchymal malignancies comprising more than 70 histological subtypes and accounting for approximately 1% of all adult cancers, with an estimated 13,400 new cases and 5140 deaths projected in the United States in 2023 alone [1,2,3]. Although STS is collectively rare, its age-related epidemiology is striking: nearly half of newly diagnosed patients are older than 65 years, and the age-adjusted incidence rises to 11.3 per 100,000 in this age group compared with 2.3 per 100,000 in younger adults [4,5]. Older patients also tend to present with biologically more aggressive disease, including a higher proportion of high-grade tumours, complex-karyotype histologies, and undifferentiated pleomorphic sarcoma [5,6]. Despite this disproportionate disease burden, patients aged ≥65 years remain markedly underrepresented in pivotal sarcoma trials—accounting for only about 12% of participants in EORTC Soft Tissue and Bone Sarcoma Group studies—which limits the generalisability of standard treatment recommendations to this population [4,7]. Compounded by age-related physiological changes, comorbidities, polypharmacy, and frailty, this evidence gap translates into undertreatment and inferior survival in routine practice [5,8,9].

Because chronological age alone poorly captures the heterogeneity of the older population, international bodies such as the International Society of Geriatric Oncology recommend structured geriatric evaluation to guide treatment decisions [10]. Validated instruments—including the G8 screening tool, comprehensive geriatric assessment, and instrumental activities of daily living—together with composite measures such as the oncological multidimensional prognostic index (Onco-MPI) have been associated with treatment tolerance and survival in older patients with advanced STS [9,11,12]. This evidence underpins the rationale for examining whether performance status, rather than chronological age, drives outcomes in this population.

For more than four decades, anthracycline-based regimens, most commonly single-agent doxorubicin, have remained the backbone of first-line systemic therapy for advanced or metastatic STS [13,14]. The randomised EORTC 62012 trial confirmed that adding ifosfamide to doxorubicin did not improve overall survival (OS) over single-agent doxorubicin (median OS, 14.3 vs. 12.8 months; hazard ratio [HR], 0.83; 95% CI, 0.67–1.03; *p* = 0.076) while substantially increasing haematological toxicity [13]. This toxicity profile is particularly problematic in older patients: doxorubicin-induced grade 4 neutropenia and febrile neutropenia occur in approximately 30% and 9% of patients, respectively, with the risk amplified in those with reduced bone-marrow reserve, comorbidities, or cardiac dysfunction [13,15,16]. Alternatives evaluated specifically in elderly patients—such as the phase II randomised comparison of trofosfamide versus doxorubicin—have failed to demonstrate superiority of less-toxic schedules while still being limited by myelosuppression and fatigue [14]. As a result, a meaningful proportion of older patients with advanced STS are deemed unfit for anthracyclines, leaving few evidence-based systemic options and a clear unmet need for tolerable, orally administered regimens that can be delivered safely in this population [5,9].

Pazopanib is an orally bioavailable, multi-targeted tyrosine kinase inhibitor of vascular endothelial growth factor receptors (VEGFR-1, -2, -3), platelet-derived growth factor receptors (PDGFR-α, -β), and KIT, exerting antitumour activity primarily through inhibition of tumour angiogenesis [17]. After favourable activity in the phase II EORTC 62043 study, in which 12-week progression-free rates exceeded the pre-specified threshold across leiomyosarcoma, synovial sarcoma, and other histological strata [18], the pivotal phase III PALETTE trial established pazopanib as the first targeted agent approved for non-adipocytic advanced STS after failure of standard chemotherapy, improving median progression-free survival (PFS) from 1.6 to 4.6 months (HR, 0.31; 95% CI, 0.24–0.40; *p* < 0.0001) without a statistically significant OS benefit [19]. In the first-line setting in older adults, the German EPAZ phase II trial subsequently demonstrated that pazopanib was non-inferior to doxorubicin in patients aged ≥60 years (median PFS, 4.4 vs. 5.3 months; HR, 1.00; 95% CI, 0.65–1.53), with a more favourable haematological profile but higher rates of hypertension and hepatic transaminase elevation [15]. Complementary phase II data in anthracycline-unfit elderly patients (mean age, 78 years) reported a clinical benefit rate of 39% at 16 weeks with manageable toxicity [20]. Several real-world series from Asian and Turkish populations have largely supported these findings, with median PFS of approximately 3–7 months and median OS of 10–14 months across unselected cohorts [21,22,23,24,25].

Despite this expanding evidence base, the published literature on pazopanib in patients aged ≥65 years with advanced STS remains limited and is dominated either by tightly selected fit-elderly populations enrolled in trials [15,20] or by mixed-age retrospective series in which the median patient age clusters around 50 years and the elderly subgroup is too small for reliable estimates [21,22,23,24,25]. Real-world effectiveness and tolerability of pazopanib in unselected older patients managed in routine oncology practice—particularly in the Turkish population, where access to alternative agents such as trabectedin or eribulin remains restricted—have not been adequately characterised. We therefore conducted a retrospective analysis of patients aged ≥65 years with advanced STS treated with pazopanib outside the clinical-trial setting. The primary endpoint was progression-free survival (PFS); secondary endpoints were overall survival (OS) and the safety profile of pazopanib in this age group. In addition, we explored baseline clinical and pathological factors associated with survival outcomes.

## 2. Materials and Methods

### 2.1. Study Design and Patient Population

This was a multicentre, retrospective cohort study conducted at four tertiary oncology centres in Turkey under the coordination of Cukurova University Faculty of Medicine. The study protocol was approved by the Cukurova University Faculty of Medicine Research Ethics Committee (Meeting No. 152, Decision No. 87; 7 February 2025), and the requirement for informed consent was waived owing to the retrospective design. All procedures were conducted in accordance with the Declaration of Helsinki and its later amendments. The study is reported in accordance with the STROBE statement for observational studies [26].

Consecutive patients were eligible if they (i) were aged ≥65 years at the time of pazopanib initiation, (ii) had a histopathologically confirmed diagnosis of advanced (locally advanced unresectable or metastatic) soft tissue sarcoma, and (iii) had received at least one dose of single-agent pazopanib between July 2010 and June 2022. Patients with adipocytic sarcomas (including liposarcoma), gastrointestinal stromal tumours, dermatofibrosarcoma protuberans, and Kaposi sarcoma were excluded, in accordance with the licensed indication of pazopanib [19]. Histological subtype was reported using the World Health Organization classification of soft tissue tumours [1], and histological grade was assigned using the FNCLCC system. Disease was staged using the AJCC/UICC 8th edition criteria.

### 2.2. Treatment and Follow-Up

Pazopanib was administered orally at a starting dose of 800 mg once daily, with dose interruptions and reductions permitted at the treating physician’s discretion in accordance with institutional protocols and the prescribing information. Treatment was continued until radiological or clinical disease progression, unacceptable toxicity, or patient withdrawal. Tumour response was assessed by the treating institution using contrast-enhanced computed tomography or magnetic resonance imaging performed at approximately 8- to 12-week intervals and categorised according to the Response Evaluation Criteria in Solid Tumours (RECIST) version 1.1 [27]. Response assessments were performed locally by each treating institution; no central or blinded independent radiological review was undertaken, and inter-centre variability in the application of RECIST 1.1 cannot be excluded.

### 2.3. Endpoints and Definitions

The primary endpoint was progression-free survival (PFS), defined as the time from pazopanib initiation to documented radiological progression or death from any cause, whichever occurred first. Secondary endpoints were overall survival (OS), defined as the time from pazopanib initiation to death from any cause, and the safety profile of pazopanib in this elderly population. The objective response rate (ORR) was defined as the proportion of patients achieving a best response of complete (CR) or partial response (PR), and the disease control rate (DCR) as the proportion achieving CR, PR, or stable disease (SD).

Treatment-emergent adverse events were extracted from medical records and graded according to the Common Terminology Criteria for Adverse Events (CTCAE) version 5.0. Because clinical documentation across participating centres systematically recorded the single most prominent treatment-emergent adverse event per patient rather than a comprehensive organ-system inventory, only the leading adverse event and its maximal grade are reported; cumulative organ-system–level adverse event burden was not captured, and this is acknowledged as a limitation.

### 2.4. Statistical Analysis

Continuous variables are summarised as median with interquartile range (IQR) and categorical variables as frequency with percentage. Group differences in baseline characteristics were assessed using the Kruskal–Wallis test for continuous variables and the χ^2^ or Fisher’s exact test for categorical variables, as appropriate. Exact binomial (Clopper–Pearson) 95% confidence intervals (CI) are reported for ORR and DCR.

Time-to-event endpoints were analysed using the Kaplan–Meier method, with 95% CIs derived from log–log transformation. Median follow-up was estimated using the reverse Kaplan–Meier method [28]. Differences between groups were tested using the log-rank test. Age was analysed as the principal stratification axis using a pre-specified three-level categorisation (65–69, 70–74, and ≥75 years), reflecting geriatric oncology conventions [10]; a binary classification (<70 vs. ≥70 years) was used in sensitivity analyses.

Cox proportional hazards regression was used to estimate hazard ratios (HR) with 95% CIs for PFS and OS. Univariate analyses tested pre-specified candidate covariates, including age (continuous and tertile), sex, ECOG performance status, FNCLCC grade, histological subtype, stage at diagnosis, liver metastasis, prior curative surgery, adjuvant chemotherapy and radiotherapy, metastasectomy, line of pazopanib therapy, and Ki-67 index. As an exploratory analysis, the association of treatment-emergent hypothyroidism, hand–foot skin reaction, and hypertension with survival outcomes was also evaluated. The multivariable model comprised a pre-specified, parsimonious set of clinically relevant covariates (sex, ECOG performance status, prior curative-intent surgery, histological subtype, and line of pazopanib therapy), selected to respect the available number of events (events-per-variable) and to avoid collinearity among prognostically overlapping variables. Notably, stage at diagnosis and adjuvant radiotherapy, although associated with outcome in univariate analysis, were not entered because their prognostic information overlaps substantially with prior curative-intent surgery; variance-inflation factors were <2.5 for all included terms. The proportional hazards assumption was verified using scaled Schoenfeld residuals (all *p* > 0.05); nevertheless, the final multivariable model was stratified by age tertile to formally account for any residual age-related heterogeneity in baseline hazards. The binary age comparison, an unstratified model with all candidate variables, and a model excluding uterine sarcomas were performed as sensitivity analyses.

All tests were two-sided, with *p* < 0.05 considered statistically significant. Data management, descriptive statistics, and univariable analyses were performed using IBM SPSS Statistics version 24.0 (IBM Corp., Armonk, NY, USA). Multivariable survival modelling and high-resolution data visualisation were conducted using Python 3.11 (packages: pandas 2.0, lifelines 0.27, statsmodels 0.14, scikit-survival 0.21).

During the preparation of this manuscript, the authors used AI-based language-editing tools solely to improve the language and readability of the text; no content, data, analysis, or interpretation was generated by these tools.

## 3. Results

### 3.1. Patient Characteristics

Between July 2010 and June 2022, a total of 109 patients aged ≥65 years with advanced soft tissue sarcoma who received pazopanib were identified across participating centres. Baseline demographic and clinical characteristics are summarised in Table 1. The median age was 70 years (IQR, 67–74; range, 65–86), and the cohort was approximately balanced for sex (55 women [50.5%] and 54 men [49.5%]). Forty-nine patients (45.0%) were aged 65–69 years, 33 (30.3%) were 70–74 years, and 27 (24.8%) were ≥75 years. An ECOG performance status of 0–1 was present in 56 patients (51.4%), while 53 (48.6%) had ECOG ≥ 2, including 13 (11.9%) with ECOG 3. ECOG performance status worsened significantly with advancing age tertile (ECOG ≥ 2 in 32.7%, 45.5%, and 81.5% of the 65–69, 70–74, and ≥75 groups, respectively; *p* < 0.001).

The most common histological subtypes were leiomyosarcoma (*n* = 42; 38.5%) and undifferentiated pleomorphic sarcoma (*n* = 19; 17.4%), with synovial sarcoma comprising 3.7% (*n* = 4); 44 patients (40.4%) had other histologies. Sixty-nine patients (63.3%) had FNCLCC grade III disease. The primary tumour arose in the extremities in 52 patients (47.7%), in the abdomen in 25 (22.9%), in the thorax in 15 (13.8%), in the uterus in 14 (12.8%), and in the scalp or head and neck in 3 (2.8%). Sixty-three patients (57.8%) presented with stage IV disease at initial diagnosis, and 42 (38.5%) had liver metastases at any time. Prior curative-intent surgery had been performed in 37 patients (33.9%), palliative surgery in 30 (27.5%), and no resection in 42 (38.5%). Seven patients (6.4%) had received adjuvant chemotherapy and 17 (15.6%) adjuvant radiotherapy. Pazopanib was administered as first-line therapy in 9 patients (8.3%), as second-line therapy in 63 (57.8%), and as third-line therapy in 37 (33.9%).

### 3.2. Tumour Response and Treatment Exposure

All 109 patients were evaluable for response. The objective response rate was 11.0% (95% CI, 5.8–18.4), comprising 3 complete responses (2.8%; 95% CI, 0.6–7.8) and 9 partial responses (8.3%; 95% CI, 3.8–15.1). Thirty-eight patients (34.9%; 95% CI, 26.0–44.6) had stable disease, and 59 (54.1%; 95% CI, 44.3–63.7) had progressive disease as the best response. The disease control rate was therefore 45.9% (95% CI, 36.3–55.7). The median duration of pazopanib exposure was 4.27 months (IQR, 2.66–6.11).

### 3.3. Progression-Free Survival

**Figure 1:**
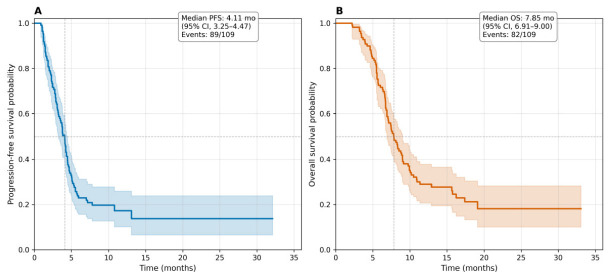
Kaplan–Meier estimates of (**A**) progression-free survival and (**B**) overall survival in the overall cohort (*n* = 109). Shaded areas represent 95% confidence intervals. Vertical tick marks indicate censored observations. The dashed lines indicate the estimated median survival and its projection onto the time axis.

**Figure 2:**
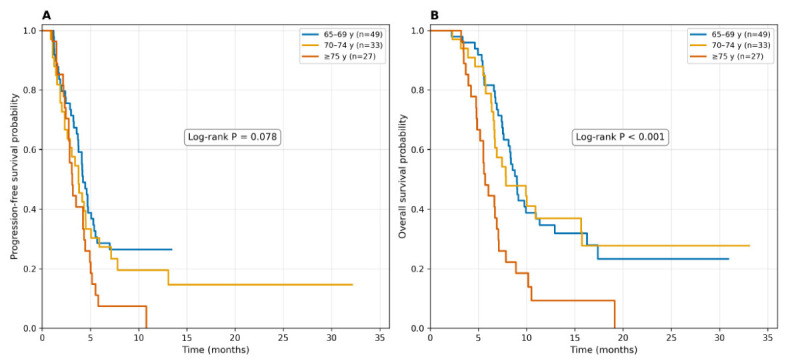
Kaplan–Meier estimates of (**A**) progression-free survival and (**B**) overall survival stratified by age tertile (65–69, 70–74, ≥75 years). Log-rank *p*-values are shown in each panel. Shaded areas represent 95% confidence intervals.

At the data cut-off, 89 of 109 patients (81.7%) had experienced disease progression over a median follow-up of 17.6 months (95% CI, 13.7–18.6; maximum, 33.1 months). The median PFS for the overall cohort was 4.11 months (95% CI, 3.25–4.47) (Figure 1A). The estimated PFS rates were 64.2% at 3 months, 22.9% at 6 months, and 17.2% at 12 months. The median PFS was 4.24, 3.78, and 3.12 months in the 65–69, 70–74, and ≥75-year age tertiles, respectively (log-rank *p* = 0.078; Figure 2A), and 4.24 and 3.52 months for patients aged <70 and ≥70 years (log-rank *p* = 0.085).

**Figure 3:**
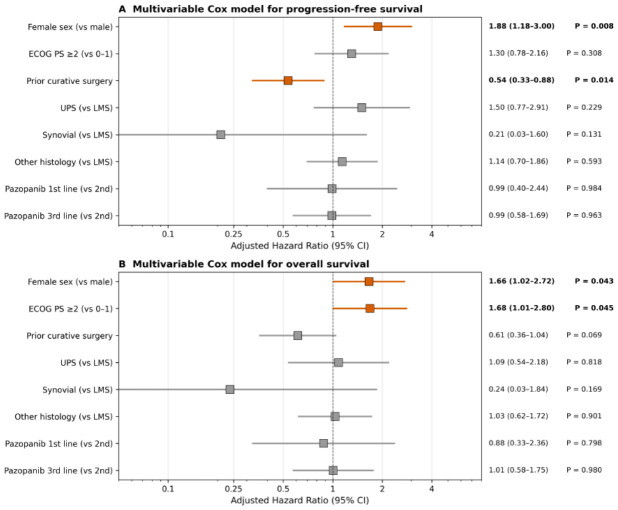
Forest plot of the multivariable Cox proportional hazards model for (**A**) progression-free survival and (**B**) overall survival, stratified by age tertile. Squares represent adjusted hazard ratios (aHR) and horizontal lines represent 95% confidence intervals. The vertical dashed line at aHR = 1.0 indicates no effect. UPS, undifferentiated pleomorphic sarcoma; ECOG PS, Eastern Cooperative Oncology Group performance status.

In univariate Cox analysis (Table 2), female sex (HR, 1.57; 95% CI, 1.03–2.39; *p* = 0.035), ECOG ≥ 2 (HR, 1.56; 95% CI, 1.03–2.38; *p* = 0.036), stage IV disease at diagnosis (HR, 1.74; 95% CI, 1.13–2.70; *p* = 0.013), and age ≥ 75 years versus 65–69 (HR, 1.64; 95% CI, 1.04–2.61; *p* = 0.035) were associated with shorter PFS, whereas prior curative surgery (HR, 0.55; 95% CI, 0.35–0.88; *p* = 0.012) and adjuvant radiotherapy (HR, 0.50; 95% CI, 0.27–0.95; *p* = 0.035) were associated with longer PFS. In the multivariable Cox model stratified by age tertile (Figure 3A and Table 2), female sex (adjusted HR [aHR], 1.88; 95% CI, 1.18–3.00; *p* = 0.008) and prior curative surgery (aHR, 0.54; 95% CI, 0.33–0.88; *p* = 0.014) remained independently associated with PFS. Exclusion of the 14 patients with primary uterine sarcomas in a sensitivity analysis strengthened, rather than attenuated, the sex effect (aHR for female sex, 2.21; 95% CI, 1.34–3.65; *p* = 0.002), indicating that the observation was not driven by primary site distribution.

### 3.4. Overall Survival

At the data cut-off, 82 of 109 patients (75.2%) had died. The median OS for the overall cohort was 7.85 months (95% CI, 6.91–9.00) (Figure 1B). The estimated OS rates were 72.5% at 6 months, 28.9% at 12 months, and 18.2% at 24 months. In contrast to PFS, a strong and statistically significant monotonic gradient in OS was observed across age tertiles: median OS was 9.00, 7.86, and 5.71 months for patients aged 65–69, 70–74, and ≥75 years, respectively (log-rank *p* < 0.001; Figure 2B). The binary <70 vs. ≥70-year comparison showed a smaller, borderline difference (9.00 vs. 6.91 months; log-rank *p* = 0.079).

Univariate predictors of shorter OS included continuous age (HR per year, 1.06; 95% CI, 1.02–1.11; *p* = 0.002), age ≥ 75 versus 65–69 (HR, 2.47; 95% CI, 1.53–3.98; *p* < 0.001), ECOG ≥ 2 (HR, 2.04; 95% CI, 1.31–3.16; *p* = 0.001), and stage IV at diagnosis (HR, 1.88; 95% CI, 1.18–2.98; *p* = 0.008) (Table 3). Prior curative surgery (HR, 0.50; 95% CI, 0.30–0.82; *p* = 0.006) and adjuvant radiotherapy (HR, 0.40; 95% CI, 0.19–0.83; *p* = 0.014) were protective. In the age-stratified multivariable model (Figure 3B and Table 3), ECOG ≥ 2 (aHR, 1.68; 95% CI, 1.01–2.80; *p* = 0.045) and female sex (aHR, 1.66; 95% CI, 1.02–2.72; *p* = 0.043) remained independently associated with OS, while prior curative surgery showed a strong protective trend that did not reach statistical significance (aHR, 0.61; 95% CI, 0.36–1.04; *p* = 0.069). The marked age-related OS gradient observed in univariable analysis was absorbed into the baseline hazard by stratification, with ECOG performance status emerging as the dominant chronological-age–independent predictor of overall survival. In a pre-specified unstratified sensitivity analysis entering all candidate variables simultaneously (Supplementary Table S1), neither stage IV disease at diagnosis (OS: HR 0.97, 95% CI 0.39–2.40, *p* = 0.94; PFS: HR 1.04, 95% CI 0.44–2.47, *p* = 0.92) nor adjuvant radiotherapy (OS: HR 0.55, 95% CI 0.17–1.77, *p* = 0.32; PFS: HR 0.75, 95% CI 0.27–2.13, *p* = 0.59) was independently associated with either endpoint, whereas the independent associations of ECOG ≥ 2 with overall survival and of female sex with progression-free survival were preserved.

### 3.5. Safety Profile

The leading treatment-emergent adverse events attributable to pazopanib are summarised in Table 4. The most commonly reported leading adverse events were hypertension (28 patients; 25.7%), fatigue/asthenia (21; 19.3%), hepatic transaminase elevation (20; 18.3%), and hypothyroidism (12; 11.0%). Hand–foot skin reaction was reported as the leading adverse event in 4 patients (3.7%). Grade ≥ 3 adverse events occurred in 30 of 109 patients (27.5%), with the most frequent being hypertension (9; 8.3%), fatigue/asthenia (5; 4.6%), hepatic transaminase elevation (4; 3.7%), and neutropenia (4; 3.7%). No grade 5 adverse events attributable to pazopanib were recorded. Treatment-emergent hypothyroidism, hand–foot skin reaction, and hypertension were not independently associated with PFS or OS in univariate analysis. Because only the single most prominent treatment-emergent adverse event per patient was documented, these frequencies represent a conservative, non-cumulative estimate; the true incidence of overlapping and low-grade toxicities could not be determined and direct numerical comparison with trial-based safety datasets is precluded.

## 4. Discussion

In this multicentre retrospective cohort of 109 patients aged ≥65 years with advanced soft tissue sarcoma treated with pazopanib in routine practice across four Turkish oncology centres, we observed a median PFS of 4.11 months and a median OS of 7.85 months, with an objective response rate of 11.0% and a disease control rate of 45.9% over a median follow-up of 17.6 months. The cohort was characterised by an unusually high burden of comorbidity and functional impairment for a treated sarcoma population—nearly half of patients (48.6%) had an ECOG performance status of ≥2, and the median age was 70 years, with one quarter aged 75 or older—reflecting the unselected real-world setting in which pazopanib is used in Turkey. While the antitumour activity of pazopanib appeared preserved across age tertiles, with median PFS values clustered between 3.12 and 4.24 months and only a borderline tertile gradient (log-rank *p* = 0.078), overall survival showed a marked monotonic decrease with advancing age, falling from 9.00 months in the 65–69-year group to 5.71 months in patients ≥ 75 years (log-rank *p* < 0.001). After stratification by age tertile in multivariable Cox analysis, an ECOG performance status ≥ 2 (aHR, 1.68; 95% CI, 1.01–2.80; *p* = 0.045) and female sex (aHR, 1.66; 95% CI, 1.02–2.72; *p* = 0.043) remained independently associated with shorter OS, while prior curative-intent surgery was independently protective for PFS (aHR, 0.54) and showed a strong protective trend for OS (aHR, 0.61; *p* = 0.069). Pazopanib was generally well tolerated, with hypertension, fatigue, hepatic transaminase elevation, and hypothyroidism being the most frequently reported treatment-emergent adverse events; grade ≥ 3 toxicity occurred in 27.5% of patients, and no fatal events were attributed to the drug.

A central observation of our study is the dissociation between PFS and OS outcomes in older patients. The median PFS of 4.11 months we observed is remarkably concordant with the 4.6 months reported in the registrational PALETTE trial [19], the 4.4 months in the pazopanib arm of the EPAZ phase II trial in patients aged ≥60 years [15], the 3.97 months in the younger (median age, 49.6 years) Turkish cohort of Karaağaç et al. [21], and the 3.67 months in the prospective phase II study of Hirbe et al. in anthracycline-unfit elderly patients (mean age, 78 years) [20]. In contrast, our median OS of 7.85 months is substantially shorter than the 12.5 months reported in PALETTE [19], the 12.3 months in the pazopanib arm of EPAZ [15], the 11.4 months in the Turkish series of Karaağaç et al. [21], the approximately 10.1 months in the multicentre Turkish study of Koca et al. [22], and the 13.8 months in the recent 552-patient Turkish Oncology Group study by Bilici et al. [23]. The convergence in PFS suggests that the on-treatment antitumour activity of pazopanib in older patients is comparable to that observed in younger and fitter populations enrolled in pivotal trials; the divergence in OS, by contrast, is most plausibly attributable to age- and comorbidity-related competing mortality and to reduced access to effective post-progression therapy in older adults, rather than to diminished pazopanib efficacy per se. This interpretation is consistent with the post hoc analysis of EPAZ by Hamacher et al. [9], which found that chronological age was not a driver of either survival or toxicity, whereas ECOG performance status and functional assessment scores (G8, IADL) were the dominant prognostic and predictive determinants. Our ORR of 11.0% and DCR of 45.9% are within the range reported across the pivotal trials (PALETTE, ORR 6%; EPAZ, ORR 12.3%) [15,19] and contemporary real-world series (Karaağaç, ORR 16.5%; Koca, ORR 20%; Alshamsan, ORR 15.5%) [21,22,29], indicating that response patterns to pazopanib are stable across age strata and care settings. For broader context, a recent retrospective study of anlotinib—another oral multikinase inhibitor—in 35 elderly patients with advanced STS (median age, 65 years) reported an ORR of 8.6%, a median PFS of 5.5 months, and a median OS of 14.3 months [30], comparable to our findings and supporting anti-angiogenic TKIs as a class-level option in this population. Histology-specific real-world data further reinforce this class effect: in a recent multicentre Turkish Oncology Group study of 51 patients with metastatic alveolar soft part sarcoma, first-line pazopanib achieved a median PFS of 27.2 months and a median overall survival of 45.0 months, substantially exceeding both chemotherapy and sunitinib controls [31].

Two findings in our cohort merit specific consideration. First, the age-related OS gradient persisted after adjustment for ECOG performance status but was attenuated, indicating that performance status mediates a substantial—but not complete—portion of the age effect on overall survival. The residual age effect is biologically plausible: with each additional decade of life, baseline organ-function reserve, immunocompetence, polypharmacy burden, and the prevalence of unmeasured comorbidities all worsen, and the probability of receiving effective post-progression therapy decreases [5,9]. Notably, ECOG was not retained as an independent predictor of PFS, whereas it was independently associated with OS; this pattern is consistent with reports from PALETTE [19], EPAZ [15], and three large real-world series [21,22,23], all of which identified performance status primarily as a survival rather than as a response predictor. Second, we observed an unexpected independent association between female sex and worse outcomes for both PFS (aHR, 1.88; 95% CI, 1.18–3.00; *p* = 0.008) and OS (aHR, 1.66; 95% CI, 1.02–2.72; *p* = 0.043), with the PFS effect strengthening rather than diminishing in a sensitivity analysis excluding the 14 patients with primary uterine sarcomas (aHR, 2.21; 95% CI, 1.34–3.65; *p* = 0.002), arguing against confounding by anatomical site. This finding contrasts with the favourable PFS association with female sex reported by Karaağaç et al. in a younger Turkish cohort (median age, 49.6 years) [21], and with the favourable OS association with female sex reported by Nakamura et al. in a Japanese cohort [25]; a unified biological explanation is not currently available. Plausible contributors include the post-menopausal endocrine and angiogenic milieu, age-related differences in sarcoma biology and histological subtype distribution, sex differences in pazopanib pharmacokinetics and tolerated dose intensity, and possible underrepresentation of male-predominant histologies such as well-tolerated synovial sarcoma in older women. Because this is, to our knowledge, the first dedicated report of pazopanib outcomes in patients aged ≥65 years to examine sex as an independent prognostic factor, we present this observation as hypothesis-generating; replication in larger elderly cohorts and prospective biomarker work are required before any clinical translation. Given the retrospective design and limited cohort size, this association may at least partly reflect residual confounding from unmeasured variables (e.g., body composition, dose intensity, pharmacokinetics, and comorbidity burden) rather than a true biological sex effect, and should not be interpreted as such in the absence of dedicated validation. Of note, neither treatment-emergent hand–foot skin reaction nor hypothyroidism was independently associated with PFS or OS in our cohort. This contrasts with the multicentre Asian study by Huang et al., in which hand–foot skin reaction was an independent predictor of better outcomes (incidence, 32%), and with the Turkish series by Karaağaç et al., which identified pazopanib-induced hypothyroidism as a favourable prognostic marker [21,32]. The discrepancy may reflect the lower incidence of these toxicities in our elderly cohort (3.7% and 11.0%, respectively, vs. 32% and 16.5% in the comparator series), reduced statistical power for detecting predictive associations of low-frequency events, and possible age-related differences in the dose intensity required to elicit these on-target adverse events. The protective trend of prior curative-intent surgery (aHR for PFS, 0.54, *p* = 0.014; aHR for OS, 0.61, *p* = 0.069) is mechanistically consistent with an oligometastatic phenotype amenable to local control, in which residual disease at the time of pazopanib initiation is biologically less aggressive than disease that has progressed continuously from initial diagnosis.

Our study has several limitations that should be considered when interpreting the findings. First, the retrospective design is intrinsically susceptible to selection and information bias, and the lack of central radiological review means that response assessments reflect institutional practice rather than blinded independent evaluation. Second, adverse event capture was limited to the single most clinically prominent treatment-emergent event per patient with its maximal CTCAE grade, rather than a comprehensive organ-system inventory; this approach almost certainly underestimates the cumulative burden of low-grade and overlapping toxicities and prevents direct numerical comparison with trial-based safety reports such as PALETTE and EPAZ [15,19]. Third, the cohort extends across a 12-year accrual period (2010–2022), during which evolving access to and use of post-progression agents (trabectedin, eribulin, doxorubicin re-challenge) may have influenced OS without a parallel effect on PFS; line-of-therapy data after pazopanib discontinuation were not systematically captured. Fourth, structured geriatric assessments (G8, IADL, Charlson Comorbidity Index)—shown in the EPAZ post hoc analysis to outperform chronological age for risk stratification [9]—were not available, limiting our ability to disentangle the contributions of biological ageing, frailty, and ECOG-captured performance status. Fifth, dose intensity over time and the proportion of patients undergoing dose reduction or interruption were not quantified, and the impact of pazopanib trough concentrations on outcomes [33] could not be assessed. Finally, the cohort is single-country and may not generalise to populations with different access to alternative second- and later-line agents. A centralised screening log quantifying the number of patients excluded at each centre and the reasons for exclusion could not be reliably reconstructed. In addition, intra-abdominal primaries—including retroperitoneal tumours, a distinct anatomical subgroup with specific surgical and prognostic considerations—were grouped as “Abdomen,” and the granular sub-site was not separately abstracted; because adipocytic histologies (the predominant retroperitoneal subtype) were excluded by design, the abdominal cases here are predominantly leiomyosarcoma and other non-adipocytic subtypes. Counterbalancing these limitations, the study has several notable strengths. To our knowledge, it represents one of the largest and most exhaustively followed real-world series focused exclusively on patients aged ≥65 years receiving pazopanib for advanced STS: with 89 of 109 PFS events (81.7%) and 82 of 109 deaths (75.2%) recorded at data cut-off over a median follow-up of 17.6 months, survival estimates are mature and supported by adequate event accrual. The pre-specified age-tertile analysis, sensitivity analyses for uterine sarcomas, and stratified multivariable modelling with formal verification of the proportional hazards assumption together support the methodological robustness of the principal conclusions.

Taken together, our findings suggest that pazopanib retains clinically meaningful antitumour activity in unselected patients aged ≥65 years with advanced soft tissue sarcoma, with response and PFS outcomes broadly comparable to those reported in younger pivotal-trial populations, but with substantially shorter overall survival driven primarily by performance status and competing age-related mortality. Two practical implications follow. First, treatment decisions in this population should be guided by performance status and—ideally—by structured geriatric assessment rather than by chronological age alone, mirroring the conclusions of EPAZ and its post hoc analysis [9,15]. Patients with ECOG 0–1 may derive clinically meaningful disease control from pazopanib, whereas those with ECOG ≥ 2 require careful counselling regarding realistic survival expectations and a low threshold for early supportive care integration. Second, the consistent PFS across age strata suggests that pazopanib should not be withheld from patients aged ≥75 years on the basis of age alone if performance status and end-organ function are acceptable. Future work should prioritise prospective validation of geriatric-assessment–driven treatment algorithms in elderly STS, exploration of dose-titration strategies analogous to that proposed by Hirbe et al. [20] in anthracycline-unfit patients, and dedicated investigation of the sex-related PFS difference we observed, ideally with concurrent assessment of pazopanib pharmacokinetics, dose intensity over time, and post-menopausal angiogenic biomarkers. Emerging inflammation-based composite biomarkers, including the pre-treatment pan-immune-inflammation value (PIV), have recently been associated with PFS and OS in pazopanib-treated STS patients [34] and warrant evaluation in dedicated elderly cohorts where systemic inflammation, sarcopenia, and frailty are tightly interlinked. Emerging molecular characterisations of sarcoma histology and of the tumour immune microenvironment may further refine prognostication and treatment selection in this setting [35,36].

## 5. Conclusions

In this multicentre retrospective cohort of 109 unselected Turkish patients aged ≥65 years with advanced soft tissue sarcoma, pazopanib produced a median progression-free survival of 4.11 months, a median overall survival of 7.85 months, an objective response rate of 11.0%, and a manageable safety profile in which 27.5% of patients experienced a leading grade ≥ 3 treatment-emergent adverse event. Progression-free survival showed only a borderline difference across age tertiles (*p* = 0.078), whereas overall survival declined monotonically with advancing age (*p* < 0.001); performance status appeared to be a more informative determinant of overall survival than chronological age; however, because structured geriatric, comorbidity, frailty, polypharmacy, dose-intensity, and post-progression treatment data were not available, this inference should be regarded as hypothesis-generating and warrants prospective validation. These findings support the use of pazopanib in older adults with adequate performance status, irrespective of age category, and underscore the value of performance-status-based and geriatric-assessment–based risk stratification over chronological-age cutoffs in routine practice.

## Figures and Tables

**Table 1:** Baseline demographic and clinical characteristics of the study cohort, stratified by age tertile.

Characteristic	Overall (*n* = 109)	65–69 y (*n* = 49)	70–74 y (*n* = 33)	≥75 y (*n* = 27)	*p*-Value
Age (years), median (IQR)	70.0 (67.0–74.0)	67.0 (65.0–68.0)	72.0 (71.0–73.0)	79.0 (76.0–81.0)	<0.001
Sex, *n* (%)					0.649
Male	54 (49.5)	22 (44.9)	17 (51.5)	15 (55.6)	
Female	55 (50.5)	27 (55.1)	16 (48.5)	12 (44.4)	
ECOG performance status, *n* (%)					<0.001
0–1	56 (51.4)	33 (67.3)	18 (54.5)	5 (18.5)	
≥2	53 (48.6)	16 (32.7)	15 (45.5)	22 (81.5)	
Histological subtype, *n* (%)					0.595
Leiomyosarcoma	42 (38.5)	18 (36.7)	13 (39.4)	11 (40.7)	
Undifferentiated pleomorphic sarcoma	19 (17.4)	10 (20.4)	3 (9.1)	6 (22.2)	
Synovial sarcoma	4 (3.7)	3 (6.1)	1 (3.0)	0 (0.0)	
Other	44 (40.4)	18 (36.7)	16 (48.5)	10 (37.0)	
FNCLCC histological grade, *n* (%)					0.999
I–II	40 (36.7)	18 (36.7)	12 (36.4)	10 (37.0)	
III	69 (63.3)	31 (63.3)	21 (63.6)	17 (63.0)	
Primary tumour site, *n* (%)					0.717
Extremity	52 (47.7)	24 (49.0)	16 (48.5)	12 (44.4)	
Abdomen	25 (22.9)	10 (20.4)	9 (27.3)	6 (22.2)	
Thoracic	15 (13.8)	6 (12.2)	5 (15.2)	4 (14.8)	
Uterine	14 (12.8)	6 (12.2)	3 (9.1)	5 (18.5)	
Scalp/head and neck	3 (2.8)	3 (6.1)	0 (0.0)	0 (0.0)	
Stage at diagnosis, *n* (%)					0.697
I–II	22 (20.2)	10 (20.4)	6 (18.2)	6 (22.2)	
III	24 (22.0)	10 (20.4)	10 (30.3)	4 (14.8)	
IV	63 (57.8)	29 (59.2)	17 (51.5)	17 (63.0)	
Liver metastasis, *n* (%)					0.516
Yes	42 (38.5)	16 (32.7)	14 (42.4)	12 (44.4)	
No	67 (61.5)	33 (67.3)	19 (57.6)	15 (55.6)	
Prior surgery, *n* (%)					0.283
Curative-intent	37 (33.9)	17 (34.7)	14 (42.4)	6 (22.2)	
Palliative	30 (27.5)	15 (30.6)	9 (27.3)	6 (22.2)	
None	42 (38.5)	17 (34.7)	10 (30.3)	15 (55.6)	
Adjuvant chemotherapy received, *n* (%)	7 (6.4)	3 (6.1)	4 (12.1)	0 (0.0)	0.162
Adjuvant radiotherapy received, *n* (%)	17 (15.6)	7 (14.3)	8 (24.2)	2 (7.4)	0.191
Metastasectomy performed, *n* (%)	8 (7.3)	4 (8.2)	4 (12.1)	0 (0.0)	0.192
Pazopanib treatment line, *n* (%)					0.076
1st line	9 (8.3)	4 (8.2)	2 (6.1)	3 (11.1)	
2nd line	63 (57.8)	25 (51.0)	17 (51.5)	21 (77.8)	
3rd line	37 (33.9)	20 (40.8)	14 (42.4)	3 (11.1)	
Ki-67 index (%), median (IQR)	38.0 (24.0–63.0)	43.0 (28.0–60.0)	36.0 (24.0–57.0)	44.0 (24.0–65.0)	0.756

Continuous variables compared with the Kruskal–Wallis test; categorical variables with the χ^2^ test or Fisher’s exact test, as appropriate. ECOG, Eastern Cooperative Oncology Group; FNCLCC, Fédération Nationale des Centres de Lutte Contre le Cancer; IQR, interquartile range. “Other” comprises: undifferentiated sarcoma NOS (*n* = 14), fibrosarcoma (*n* = 7), malignant mesenchymoma (*n* = 5), malignant peripheral nerve sheath tumour (*n* = 5), myxofibrosarcoma (*n* = 4), angiosarcoma (*n* = 3), epithelioid sarcoma (*n* = 2), epithelioid haemangioendothelioma (*n* = 1), rhabdomyosarcoma (*n* = 1), alveolar soft-part sarcoma (*n* = 1), and desmoplastic small round-cell tumour (*n* = 1).

**Table 2:** Univariable and multivariable Cox proportional hazards analyses for progression-free survival.

Variable	Univariable HR (95% CI)	*p*	Multivariable HR (95% CI) ^a^	*p*
Age (per 1-year increase)	1.03 (1.00–1.07)	0.072	*Stratification variable*	—
Age 70–74 (vs. others)	1.01 (0.64–1.59)	0.980	*Stratification variable*	—
Age ≥ 75 (vs. others)	1.64 (1.04–2.61)	0.035	*Stratification variable*	—
Female sex (vs. male)	1.57 (1.03–2.39)	0.035	1.88 (1.18–3.00)	0.008
ECOG PS ≥ 2 (vs. 0–1)	1.56 (1.03–2.38)	0.036	1.30 (0.78–2.16)	0.308
FNCLCC grade III (vs. I–II)	1.10 (0.72–1.70)	0.652	*Not included*	—
Stage IV at diagnosis (vs. I–III)	1.74 (1.13–2.70)	0.013	*Not included*	—
Liver metastasis (present vs. absent)	1.05 (0.68–1.60)	0.834	*Not included*	—
Prior curative surgery (vs. none/palliative)	0.55 (0.35–0.88)	0.012	0.54 (0.33–0.88)	0.014
Adjuvant chemotherapy (yes vs. no)	0.66 (0.27–1.63)	0.367	*Not included*	—
Adjuvant radiotherapy (yes vs. no)	0.50 (0.27–0.95)	0.035	*Not included*	—
Metastasectomy (yes vs. no)	0.52 (0.21–1.28)	0.156	*Not included*	—
UPS (vs. leiomyosarcoma)	1.15 (0.67–1.98)	0.608	1.50 (0.77–2.91)	0.229
Synovial sarcoma (vs. leiomyosarcoma)	0.16 (0.02–1.16)	0.069	0.21 (0.03–1.60)	0.131
Other histology (vs. leiomyosarcoma)	1.10 (0.72–1.69)	0.645	1.14 (0.70–1.86)	0.593
Pazopanib 1st line (vs. 2nd)	0.59 (0.26–1.36)	0.215	0.99 (0.40–2.44)	0.984
Pazopanib 3rd line (vs. 2nd)	0.93 (0.59–1.46)	0.751	0.99 (0.58–1.69)	0.963
Ki-67 (per 1% increase)	1.00 (0.99–1.01)	0.906	*Not included*	—

Hazard ratios derived from Cox proportional hazards regression. ^a^ Multivariable model stratified by age tertile (65–69, 70–74, ≥75 years); therefore, age categories are not estimated in the multivariable column. Italicised entries denote terms not estimated in the multivariable model. In the univariate column, each of the 70–74 and ≥75 tertiles was tested against the remainder of the cohort as a one-vs-rest binary covariate. CI, confidence interval; ECOG PS, Eastern Cooperative Oncology Group performance status; FNCLCC, Fédération Nationale des Centres de Lutte Contre le Cancer; HR, hazard ratio; UPS, undifferentiated pleomorphic sarcoma.

**Table 3:** Univariable and multivariable Cox proportional hazards analyses for overall survival.

Variable	Univariable HR (95% CI)	*p*	Multivariable HR (95% CI) ^a^	*p*
Age (per 1-year increase)	1.06 (1.02–1.11)	0.002	*Stratification variable*	—
Age 70–74 (vs. others)	0.77 (0.47–1.25)	0.288	*Stratification variable*	—
Age ≥ 75 (vs. others)	2.47 (1.53–3.98)	<0.001	*Stratification variable*	—
Female sex (vs. male)	1.48 (0.95–2.29)	0.082	1.66 (1.02–2.72)	0.043
ECOG PS ≥ 2 (vs. 0–1)	2.04 (1.31–3.16)	0.001	1.68 (1.01–2.80)	0.045
FNCLCC grade III (vs. I–II)	0.92 (0.59–1.45)	0.732	*Not included*	—
Stage IV at diagnosis (vs. I–III)	1.88 (1.18–2.98)	0.008	*Not included*	—
Liver metastasis (present vs. absent)	1.12 (0.72–1.74)	0.622	*Not included*	—
Prior curative surgery (vs. none/palliative)	0.50 (0.30–0.82)	0.006	0.61 (0.36–1.04)	0.069
Adjuvant chemotherapy (yes vs. no)	0.39 (0.12–1.24)	0.110	*Not included*	—
Adjuvant radiotherapy (yes vs. no)	0.40 (0.19–0.83)	0.014	*Not included*	—
Metastasectomy (yes vs. no)	0.44 (0.16–1.19)	0.106	*Not included*	—
UPS (vs. leiomyosarcoma)	1.06 (0.60–1.85)	0.850	1.09 (0.54–2.18)	0.818
Synovial sarcoma (vs. leiomyosarcoma)	0.20 (0.03–1.46)	0.113	0.24 (0.03–1.84)	0.169
Other histology (vs. leiomyosarcoma)	1.06 (0.68–1.64)	0.801	1.03 (0.62–1.72)	0.901
Pazopanib 1st line (vs. 2nd)	0.56 (0.23–1.39)	0.213	0.88 (0.33–2.36)	0.798
Pazopanib 3rd line (vs. 2nd)	0.87 (0.54–1.38)	0.545	1.01 (0.58–1.75)	0.980
Ki-67 (per 1% increase)	1.00 (0.99–1.01)	0.618	*Not included*	—

Hazard ratios derived from Cox proportional hazards regression. ^a^ Multivariable model stratified by age tertile (65–69, 70–74, ≥75 years); therefore, age categories are not estimated in the multivariable column. Italicised entries denote terms not estimated in the multivariable model. In the univariate column, each of the 70–74 and ≥75 tertiles was tested against the remainder of the cohort as a one-vs-rest binary covariate. Abbreviations in Table 2.

**Table 4:** Leading treatment-emergent adverse events attributable to pazopanib (*n* = 109).

Adverse Event	Any Grade *n* (%)	Grade 1 *n*	Grade 2 *n*	Grade 3 *n*	Grade 4 *n*	Grade ≥ 3 *n* (%)
Hypertension	28 (25.7)	7	12	8	1	9 (8.3)
Fatigue/asthenia	21 (19.3)	10	6	3	2	5 (4.6)
Hepatic transaminase elevation	20 (18.3)	7	9	4	—	4 (3.7)
Hypothyroidism	12 (11.0)	—	10	1	1	2 (1.8)
Diarrhoea	9 (8.3)	1	6	2	—	2 (1.8)
Neutropenia	6 (5.5)	1	1	3	1	4 (3.7)
Hand–foot skin reaction	4 (3.7)	—	2	2	—	2 (1.8)
Thrombocytopenia	4 (3.7)	1	1	1	1	2 (1.8)
Nausea	3 (2.8)	—	3	—	—	0 (0.0)
Proteinuria	2 (1.8)	1	1	—	—	0 (0.0)
**Patients with a leading adverse event recorded (by design)**	**109 (100.0)**	—	—	—	—	**30 (27.5)**

Adverse events were graded according to the Common Terminology Criteria for Adverse Events (CTCAE) version 5.0. Clinical documentation systematically recorded only the single most clinically prominent treatment-emergent adverse event per patient and its maximal grade; cumulative organ-system–level adverse event burden was not captured (see Section 2.3). No grade 5 (fatal) adverse events attributable to pazopanib were recorded. The denominator for all percentages is the total cohort (*n* = 109). By design, exactly one leading treatment-emergent adverse event was recorded per patient; the figure of 109 (100%) therefore reflects this capture method and does not indicate that all patients experienced clinically significant toxicity. Bold indicates the summary row.

## Data Availability

The de-identified data supporting the findings of this study are available from the corresponding author upon reasonable request and subject to institutional and ethical approvals.

## Supplementary Materials

The following supporting information can be downloaded at: https://www.mdpi.com/article/10.3390/jcm15124803/s1, Table S1: Unstratified multivariable Cox proportional hazards model including all pre-specified candidate variables for overall survival and progression-free survival (sensitivity analysis).
