# Supplementary material for: Efficacy and Tolerability of Pazopanib in Elderly Patients with Advanced Soft Tissue Sarcoma: A Multicentre Real-World Study from Turkey
Source: J Clin Med. 2026 Jun 20;15(12):4803. doi: 10.3390/jcm15124803 (PMC13302015; doi:10.3390/jcm15124803)
Supplement: Supplementary file 1 [file jcm-15-04803-s001.zip › jcm-4353497-supplementary.pdf]

Table S1. Unstratified multivariable Cox proportional hazards model including all pre-specified candidate variables for overall survival and progression-free survival (sensitivity analysis).

	**Overall survival**		**Progression-free survival**	
**Variable**	**aHR (95% CI)**	***P***	**aHR (95% CI)**	***P***
Age (per 1-year increase)	1.04 (0.99–1.09)	0.088	1.02 (0.98–1.07)	0.274
Female sex (vs male)	1.50 (0.88–2.57)	0.137	**1.80 (1.09–2.97)**	**0.022**
ECOG PS ≥2 (vs 0–1)	**1.89 (1.10–3.27)**	**0.022**	1.39 (0.83–2.34)	0.210
FNCLCC grade III (vs I–II)	0.74 (0.34–1.66)	0.470	0.94 (0.45–1.96)	0.875
Stage IV at diagnosis (vs I–III)	0.97 (0.39–2.40)	0.943	1.04 (0.44–2.47)	0.921
Liver metastasis (present vs absent)	1.15 (0.69–1.90)	0.594	1.04 (0.65–1.68)	0.864
Prior curative surgery (vs none/palliative)	0.68 (0.23–2.01)	0.486	0.61 (0.22–1.70)	0.343
Adjuvant chemotherapy (yes vs no)	1.38 (0.30–6.42)	0.681	2.03 (0.57–7.19)	0.273
Adjuvant radiotherapy (yes vs no)	0.55 (0.17–1.77)	0.317	0.75 (0.27–2.13)	0.594
Metastasectomy (yes vs no)	0.40 (0.13–1.19)	0.099	0.64 (0.24–1.72)	0.372
UPS (vs leiomyosarcoma)	1.03 (0.51–2.07)	0.932	1.40 (0.73–2.69)	0.309
Synovial sarcoma (vs leiomyosarcoma)	0.26 (0.03–2.10)	0.205	0.18 (0.02–1.41)	0.101
Other histology (vs leiomyosarcoma)	1.05 (0.60–1.81)	0.872	1.06 (0.63–1.77)	0.838
Pazopanib 1st line (vs 2nd)	0.76 (0.26–2.18)	0.610	0.86 (0.33–2.22)	0.754
Pazopanib 3rd line (vs 2nd)	1.17 (0.65–2.12)	0.595	1.09 (0.62–1.90)	0.771
Ki-67 (per 1% increase)	1.00 (0.98–1.02)	0.983	1.00 (0.99–1.02)	0.854

Hazard ratios were derived from a single multivariable Cox proportional hazards model entering all pre-specified candidate variables simultaneously, without stratification by age tertile (in contrast to the age-stratified primary models in Table 2 and Table 3). Age was modelled as a continuous covariate (per 1-year increase). The analysis was based on 82 deaths (OS) and 89 progression events (PFS); given the number of candidate covariates, the events-per-variable ratio is below the conventional threshold of 10, and this exploratory model is therefore intended solely to assess the robustness of the primary findings rather than for formal inference. In this model, neither stage IV disease at diagnosis nor adjuvant radiotherapy was independently associated with either endpoint; ECOG PS ≥2 remained independently associated with overall survival and female sex with progression-free survival, and all effect directions were preserved relative to the primary models.

Bold values denote $P < 0.05$. aHR, adjusted hazard ratio; CI, confidence interval; ECOG PS, Eastern Cooperative Oncology Group performance status; FNCLCC, Fédération Nationale des Centres de Lutte Contre le Cancer; UPS, undifferentiated pleomorphic sarcoma.
